# The structure stability of negative symptoms: longitudinal network analysis of the Brief Negative Symptom Scale in subjects with schizophrenia

**DOI:** 10.1192/j.eurpsy.2023.593

**Published:** 2023-07-19

**Authors:** E. Caporusso, G. M. Giordano, A. Mucci, P. Rucci, F. Sanmarchi, L. Giuliani, A. Perrottelli, P. Pezzella, P. Bucci, P. Rocca, A. Rossi, A. Bertolino, S. Galderisi, M. Maj

**Affiliations:** ^1^Department of Psychiatry, University of Campania “Luigi Vanvitelli”, Naples; ^2^Department of Biomedical and Neuromotor Sciences, University of Bologna, Bologna; ^3^Department of Neuroscience, Section of Psychiatry, University of Turin, Turin; ^4^Section of Psychiatry, Department of Biotechnological and Applied Clinical Sciences, University of L’Aquila, L’Aquila; ^5^Department of Basic Medical Science, Neuroscience and Sense Organs, University of Bari ‘Aldo Moro’, Bari, Italy

## Abstract

**Introduction:**

Negative symptoms (NS) represent an unmet need of treatment in schizophrenia (SCZ). As a result, these symptoms pose a significant burden on patients, their families, and the health care system. In the last decade, the conceptualization model that has received the most support from the literature has described 2 domains of NS: the expressive deficit (EXP), which includes blunted affect and alogia, and the motivational deficit (MAP), which includes avolition, asociality, and anhedonia. However, different confirmatory factor-analytic studies suggest that the bi-dimensional model may not capture the complexity of this construct, which could be better defined by the 5-factor model. To date no study exploiting innovative tools and state of the art assessment instruments has yet been conducted to evaluate the NS structure stability over time.

**Objectives:**

The aim of this study was to investigate the stability of the latent structure of NS in subjects with SCZ.

**Methods:**

NS were assessed in 612 subjects with SCZ using the Brief Negative Symptom Scale (BNSS) at the baseline and after 4-year follow-up. A network invariance analysis was conducted for the data collected longitudinally.

**Results:**

Results showed that the BNSS’ items aggregated to form 5 distinct domains (avolition, asociality, blunted affect, alogia and anhedonia). The result of the network invariance test indicated that the network structure remained unchanged over time (network invariance test = 0.13; p = 0.169) while its overall strength decreased significantly (6.28 baseline, 5.79 at follow-up; global strength invariance test = 0.48; p = 0.016).

**Conclusions:**

The results of this study show how the construct of NS can be better explained by the 5 individual negative symptoms and that this model is almost stable over time. Therefore the 2-dimensional model may be insufficient to describe the characteristics of NS. This data is of important relevance with consequent implications in the study of pathophysiological mechanisms and the development of targeted treatments for NS.

**Disclosure of Interest:**

None Declared

